# Multilevel model in the identification of behavioral and structural risk factors for HIV: integrative review

**DOI:** 10.1590/0034-7167-2021-0853

**Published:** 2022-12-16

**Authors:** Juliana Kelly Batista da Silva, Jamira Martins dos Santos, William Caracas Moreira, Renata Olívia Gadelha Romero, Oriana Deyze Correia Paiva Leadebal, Jordana de Almeida Nogueira

**Affiliations:** I Universidade Federal da Paraíba. João Pessoa, Paraíba, Brazil

**Keywords:** HIV, Multilevel Analysis, Behavior, Risk, Models Structural., VIH, Análisis Multinivel, Comportamiento, Riesgo, Modelos Estructurales., HIV, Análise Multinível, Comportamento, Risco, Modelos Estruturais.

## Abstract

**Objectives:**

to investigate studies that adopted the multilevel analysis model to identify behavioral and structural risk factors associated with HIV infection.

**Methods:**

an integrative review of the literature with studies available in full, obtained from EMBASE, CINAHL, Pubmed, and *Scopus*, whose selected descriptors were the indexed terms: “HIV”, “multilevel analysis” and “behavior”.

**Results:**

the search resulted in 236 studies. Out of these, ten studies comprised the sample. Economic disadvantage, neighborhood characteristics, housing instability, incarceration, transactional sex, multiple partners, substance abuse, and age at first intercourse were classified as structural and behavioral risk factors for HIV. Reduced socioeconomic disadvantage, provision of housing stability, and condom use were associated with protective factors for HIV exposure.

**Conclusions:**

by applying the multilevel model in risk factor research studies, it was possible to identify the structural and behavioral elements of HIV risk.

## RESUMO

**Objetivos:**

investigar estudos que adotaram o modelo de análise multinível na identificação de fatores de risco comportamentais e estruturais, que estão associados a infecção pelo HIV.

**Métodos:**

revisão integrativa da literatura com estudos disponíveis na íntegra, obtidos nas bases EMBASE, CINAHL, Pubmed e *Scopus*, cujos descritores selecionados foram os termos constantes: “*HIV”, “multilevel analysis”, “behavior”*.

**Resultados:**

a pesquisa resultou em 236 artigos. Destes, dez artigos compuseram a amostra. Desvantagem econômica, características de vizinhança, instabilidade habitacional, encarceramento, sexo transacional, múltiplos parceiros, abuso de substâncias e idade da primeira relação sexual foram classificados como fatores de risco estruturais e comportamentais ao HIV. Redução da desvantagem socioeconômica, fornecimento de estabilidade habitacional e uso de preservativos foram associados a fatores de proteção à exposição ao HIV.

**Conclusões:**

com a aplicabilidade do modelo multinível nos estudos de investigação de fatores de risco, foi possível identificar os elementos estruturais e comportamentais de risco ao HIV.

## RESUMEN

**Objetivos:**

investigar estudios que adoptaron el modelo del análisis multinivel para identificar los factores de riesgo comportamentales y estructurales asociados al VIH.

**Método:**

es una revisión bibliográfica integradora con estudios disponibles en su totalidad, obtenidos de las bases de datos EMBASE, CINAHL, Pubmed y *Scopus*, cuyos descriptores fueron los términos: “HIV”, “*multilevel analysis*”, “*behavior*”.

**Resultados:**

diez artículos, de los 236 encontrados, formaron parte de la muestra. Desventajas económicas, características del vecindario, inestabilidad habitacional, encarcelamiento, sexo transaccional, parejas múltiples, abuso de sustancias y edad de la primera relación sexual se destacaron como factores de riesgo estructurales y comportamentales del VIH. La reducción de desventajas socioeconómicas, la provisión de estabilidad habitacional y el uso de preservativos están asociados a la protección contra el VIH.

**Conclusiones:**

al aplicar el modelo multinivel en los estudios de investigación de los factores de riesgo, fue posible identificar los elementos estructurales y comportamentales del riesgo del VIH.

## INTRODUCTION

In 2020, 37.7 million people were living with the Human Immunodeficiency Virus (HIV) worldwide. Out of these, 36 million were adults, and 1.7 million were children (0-14 years old). Even though the last ten years have seen a 31% decline in the incidence rate, 1.5 million people contracted the virus in the last year^(1)^.

More specifically, in Brazil, between 2007 and June 2020, 237,551 new cases were reported in the Information System of Notifiable Diseases (Sistema de Informação de Agravos de Notificação - SINAN), 69.4% in men and 30.6% in women. Regarding the age groups, there was a higher HIV infection distribution between the ages of 20 and 34 (52.7%). As for Acquired Immunodeficiency Syndrome (AIDS) data, in 2019, young adults aged 20 to 29 years were the population most affected^(2)^.

The epidemiological scenario still reflects the disparities that underlie HIV infection. Contextual, structural, and behavioral factors, as well as elements that elucidate and reinforce the basis for understanding HIV outcomes, must be examined^(3)^.

Behavioral and structural factors can be associated with the institutional condition in which the individual is socially organized. Considering that it is not only determined by instincts, the involvement in high-risk sexual behavior can also be determined by the community characteristics to which the individual belongs^(4)^.

The complexity in understanding the determinants of sexual risk behavior for HIV infection is exemplifiable by involving multifactorial, complex, and intersecting levels, which are mostly loaded with multiple individual and social experiences. A cohort study conducted in South Africa provided evidence for the likelihood of HIV infection being related to gender, behavioral, social, communication, and HIV prevalence factors in the territory^(4-5)^.

In the current framework regarding HIV prevention and control actions, emphasis is placed on the behavioral axis of the individual, generating specific interventions with little focus on the individual’s structural aspects. It is necessary to consider factors associated with substantial changes in the family, community, society, and public policies for HIV prevention and control efforts to be effective^(6-7)^. A study points out that the high prevalence of HIV is related to difficult access to services, limitations in prevention actions, and fragility in the structural support of individuals and the community^(8)^.

The coordinated knowledge of multiple behavioral and structural levels is essential in designing effective interventions to reduce HIV epidemic advances^(4-5,9)^. Multilevel models are characterized by producing results with significant effects at each study level, elaborating specific grounds for better intervention planning, and providing subsidies regarding the evidence justifying the need to consider the context or social setting in which the phenomena occur^(4,10-11)^.

Associating individuals in the same community or country and the effects of individual attributes and social context characteristics are possible with the model’s applicability, as well as the coordinated approach development, and are therefore not typical of a single knowledge field^(10-11)^. In this context, the articulation level study that addresses the precepts associated with structure and behavior predisposed to HIV infection is necessary for multifactorial approaches to addressing the HIV epidemic. Thus, associating the individual’s social context with these ecological scores reflects a better comprehension of the factors associated with HIV^(4,12)^.

Despite epidemiological trends presented, we note that well-established risk levels can quantify the effects of current approaches implemented in health services, with awareness of new approaches and modeling factors associated with a broader understanding of the mechanisms impacting HIV transmission^(3,7,12)^.

## OBJECTIVES

To investigate studies that have adopted the multilevel analysis model in identifying behavioral and structural risk factors associated with HIV infection.

## METHODS

This is an Integrative Review of the Literature (IRL) which present the best available evidence on the intervention analyzed, being characterized as a scientific method that allows a pragmatic and evidence-based instrumentation of its findings^(13)^. For its operationalization, the following steps were taken: identifying the topic and selecting the research question for the integrative review; selecting the databases; specifying the criteria for inclusion and exclusion of studies (sample selection); defining the information to be extracted from the selected studies (study categorization); evaluating the studies included in the integrative review (results analysis); interpreting the results and presenting the review (knowledge synthesis)^(14-15)^.

The research question was structured from the PICOS strategy protocol, which represents an acromion: Participants, Intervention, Comparison, Outcome, and Study design, considering: P - youth and adults; I - multilevel analysis; C - behavioral and structural; O - HIV, and S - a meta-analysis of multiple controlled studies; individual with experimental or quasi-experimental design; time series or case-control; descriptive correlational research^(14-15)^. Therefore, the following question was formulated: *does the use of the multilevel analysis model enable the identification of behavioral and structural risk factors for HIV infection?*

**Figure 1:**
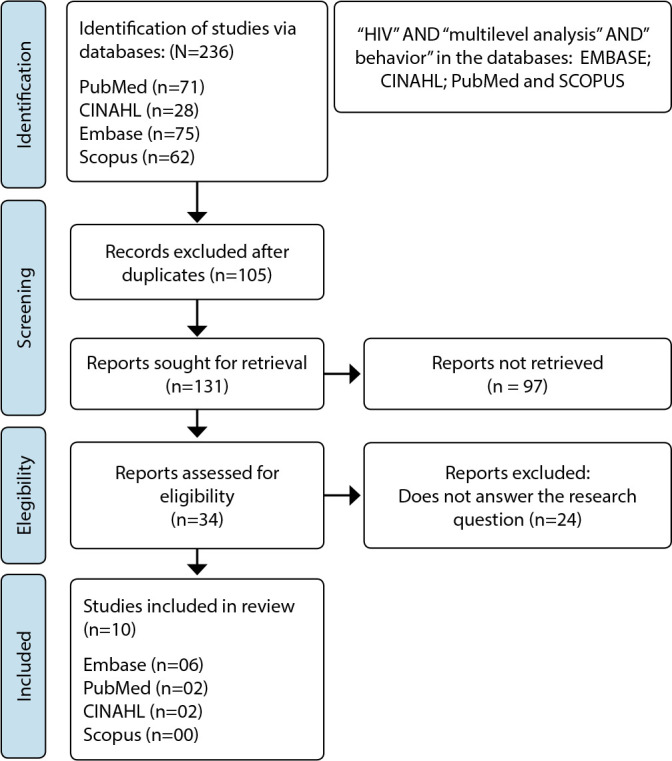
PRISMA flowchart describing the search and selection of studies^(17)^

The electronic search was conducted in the databases during May and June 2021 through the following portals: EMBASE, Cumulative Index to Nursing and Allied Health Literature (CINAHL), National Library of Medicine and National Institutes of Health (PubMed), and Scopus. To ensure the careful search steps, the descriptors selected were the terms in the Medical Subject Headings (MeSH) with Acronym: “HIV”, “multilevel analysis”, “behavior”. The AND operator was used to combine blocks of research and/or distinct concepts^(16)^. The review search process followed the recommendations of the Preferred Reporting Items for Systematic Reviews and Meta-Analysis (PRISMA)^(17)^, according to Figure 1.

For sample selection, the following inclusion criteria were observed: articles published in Portuguese, English, and Spanish, available in full, with interventions associated with the study object and types of studies described in the “study design” step of the PICOS strategy, published in the period between January 2010 and December 2020. We emphasize that the period was selected due to the availability of publications on the research question^(14-15,17)^.

Dissertations/theses, editorials, expert opinions, letters to the editor, case studies, and case report level or data obtained from reputable authorities based on clinical competence or expert committee opinion, including interpretations of information not based on research and articles in other languages, were excluded.

For the initial screening of abstracts, titles, and duplicate studies removal, the Rayyan tool was used to assist in the review automation, maintaining concealment among reviewers, and incorporating a high usability level^(18)^. EndNote software was used for reference management^(16)^. In order to ensure transparency, clarity, and traceability of the selection process and consequently avoid selection bias, three researchers acted independently, and the evidence for each axis of the PICOS strategy was assessed, associated with each possible recommendation, and there was consensus on the selections. Disagreements and conflicts were resolved through continued selections, and revision of recommendations to provide precision/qualification included in the original research question^(16,18-19)^.

The search resulted in 236 studies, out of which 105 were excluded for presenting duplicates, followed by selective reading of the titles and abstracts that resulted in 131 studies. Among these, 97 studies were excluded for not fulfilling the PICOS strategy; thus, 34 studies were selected. In this last phase, the pre-selected studies (34) were read in full, listing the research findings with the evaluation of the content and its relevance in contributing to the Outcome stage for understanding the phenomenon studied, resulting in the inclusion of 10 studies from the following databases: EMBASE (six); PubMed (two) and CINAHL (two)^(15)^.

After reading the included studies, for result analysis, a data collection instrument was prepared to contain the following variables: title of the article, authors, place and year of the study, journal and objectives, study type, age range of the study population, research design, multilevel model elements and results/outcomes^(14,16)^.

For data categorization and presentation, the results were presented descriptively through a summary table to highlight relevant information from the selected studies and the multilevel model associated with HIV infection. The risk factors evidenced in the selected texts were grouped in the category “HIV risk factors” (Chart 1).

**Chart 1:** Summary table with distribution of selected studies, according to title, year, country, design, age group, multilevel model and HIV risk factors, 2010-2020

Title/Study number	Year/Country	Design/population	Multi-level model elements	HIV risk factors
A multilevel analysis of the determinants of high-risk sexual behaviour in Sub-Saharan Africa (E1)^(4)^	2012 Sub-Saharan Africa	Cross-sectional study (n=144,983); with women aged 15-49 and men aged 15-54/59	Factors related to micro- and macro-level involvement in high-risk sexual behaviors and sexual exposure	Involvement with multiple sexual partners and early initiation of sexual activity; exposure to media;
A qualitative study of young men who have sex with men and multilevel factors related to hiv risks in Malaysia (E2)^(21)^	2018 Malaysia	Qualitative study (n=24); with young people aged 18-25 years;	Explored the multi-level domains: perception and acceptance by family, friends, and society as men who have sex with men; gender roles; relationships; condom use; HIV and HIV testing	Lack of adequate sex education in schools; difficulty in buying condoms; lack of easily accessible and confidential tests in testing clinics; inability to reconcile sexuality with cultural and religious backgrounds; and social homophobia;
A Multilevel Analysis of Neighborhood Socioeconomic Disadvantage and Transactional Sex with Casual Partners Among Young Men Who Have Sex with Men Living in Metro Detroit(E3)^(22)^	2016 USA	Cross-sectional study (n= 319), with young people aged 18 to 29 years;	Level 1: transactional sex with casual partner; HIV testing and status; substance use, demographic characteristics; Level 2: neighborhood and neighborhood economic disadvantage;	Young people living at high socioeconomic disadvantage; involvement in active transsexual with casual partners; socioeconomic neighborhood is associated with HIV risk behaviors
Relationships between neighborhood characteristics and current STI status among HIV-infected and HIV-uninfected women living in the Southern USA: a cross-sectional multilevel analysis (E4)^(23)^	2017 USA	Cohort study (n=737) with women aged 25 to 60 years;	Associations between neighborhood characteristics and associations through Socioecological Framework analysis and controlled multilevel analysis;	Area level with higher social disorder (higher violent crime, vacant housing, poverty, STI prevalence); area level with social disadvantage (more alcohol outlets, renter-occupied housing);
Social disequilibrium and the risk of HIV acquisition: a multilevel study in Rural KwaZulu-Natal Province, South Africa (E5)^(5)^	2017 South Africa	Cohort Study (n= 17,376), with men aged 15-54 years, and women aged 15-49 years;	Multilevel survival models to examine social determinants, HIV prevalence, and individual determinants	Access points with higher intensity of neighborhood migration among men; in women, higher intensity of neighborhood migration, youth, sexual initiation, contraception, circumcision, and social determinants; neighborhood characteristics are attributable to HIV risk;
Spatial patterns and associated factors of HIV Seropositivity among adults in Ethiopia from EDHS 2016: a spatial and multilevel Analysis (E6)^(24)^	2020 Ethiopia	Cross-sectional study (n=25,774) adults aged 15 to 59 years;	Analysis of spatial heterogeneity, and multilevel logistic, to identify factors associated with HIV	Place of residence was associated with HIV seropositivity; high age; individual sex, individuals exposed to high levels of media exposure, urban residence and smaller family size;
Sex ratio, poverty, and concurrent partnerships among men and women in the United States: a multilevel analysis (E7)^(25)^	2013 United States of America	Cross-sectional study (n= 12,571) youth and adults aged 15 to 44 years;	Simultaneous analysis over the past 12 months regarding the County’s sex ratio (among the participants racial and ethnic group), percentage in poverty (among the respondent’s racial and ethnic group), and violent crime rate;	Age at first intercourse and substance abuse were associated with concurrency; sexual network patterns such as concurrent partnerships are critical determinants of HIV spread in the population; black men with a low sex ratio and high incarceration rates were more likely to have multiple partners;
Structural Efects on HIV Risk Among Youth: A Multi‑level Analysis (E8)^(26)^	2018 Colombia	Cross-Sectional Study (n=1,793) with young people aged 12 to 24 years.	To confirm associations between: HIV status; concentrated community disadvantage; HIV structural stigma, sexual and gender minority structural stigma; community support for youth; neighborhood opportunity structures; early HIV stigma; HIV risk sexual relationships;	Disadvantage of concentrated community; residing in a state with failures to confer legal protections for sexual practices and gender minorities on youth participation in pro-social age activities was significant;
The relationship between economic deprivation, housing instability and transactional sex among women in North Carolina (HPTN 064) (E9)^(27)^	2019 United States of America	Cohort study (n= 2,099) with women between 18 and 44 years old;	Relationship between area and individual level measures and transactional sex; and the relationship between transactional sex as an exposure and HIV risk related characteristics	Increased transactional sex was associated with food insecurity; housing instability, substance abuse, and partner incarceration; partner in incarceration was associated with HIV risk;
A multilevel analysis of the determinants and cross-national variations of HIV seropositivity in sub-Saharan Africa: Evidence from the DHS (E10)^(10)^	2011 Sub-Saharan Africa	Cross-sectional study (n= 174,592), with youth and adults aged 15-44 years;	Applied multilevel logistic regression models to explore individual and regional contextual and country levels of factors associated with HIV seropositivity risk;	Socioeconomic factors linked to transactional sex and vulnerability; women in their 30s, elementary school education; low media exposure; female-headed households;

The points of the essential elements for the representation of behavioral and structural factors associated with the HIV infection risk in the selected articles were presented by grouping the studies into two categories: *structural and behavioral risk factors and structural and behavioral protective factors* ^(20)^. We chose to include the findings associated with lower exposure to HIV for a better representation of the results/outcomes (Chart 2 and 3). These categories were produced from the synthesis of each study included in the integrative review^(13-14,20)^.

**Chart 2:** Risk factors for HIV indicated in the studies in the *structural and behavioral risk factors category*

*Structural risk factors*	*Behavioral risk factors*
Socioeconomic Disadvantage^(10,22)^ and Social Disorder^(23,26)^	Transactional sex^(10,22,27)^
Neighborhood characteristics^(5,22-23)^	Multiple partners^(5,25)^
Media exposure^(4,24)^	Substance abuse^(23,25)^
Lack of sex education^(21)^, difficulty in buying condoms^(21)^	Condom Accessibility^(21)^
Lack of testing^(21)^	Age at first sexual intercourse^(23,25)^
Migration^(5)^	Circumcision^(5)^
Housing instability^(24,27)^	Individual Sex^(24)^
Imprisonment^(25,27)^	Lack of legal protections for sexual practices^(26)^

**Chart 3:** Protective factors for exposure to HIV pointed out in the studies in the *category structural and behavioral protective factors*

*Structural Protection Factors*	*Behavioral protective factors*
Accessibility to voluntary testing^(4,21)^	Condom use^(2,26)^
Funding from Non-Governmental Organizations^(21)^	Individual autonomy and attitudes to sexual practices^(4)^
Reduce socioeconomic disadvantage, and provide housing stability^(22,27)^	Alternative options to transactional sex^(22)^
Explore vulnerable neighborhoods^(23)^	Temporality in relationships^(23)^
Exploring mechanisms between social connections and HIV acquisition^(5)^	Increased use of health resources^(26)^
Community support, engagement in community activities^(26)^	Behavioral interventions^(25)^

## RESULTS

From the analysis of the selected texts, Chart 1 presents the results regarding the title, year, country, design, age group studied, elements used to apply the multilevel model and HIV risk factors. Regarding the publication year of the studies, two articles were published in 2018 and two in 2017. Furthermore, one study was observed annually, distributed among the years 2011, 2012, 2013, 2016, 2019, and 2020.

International journals accounted for 100% of the publications, with the Aids and behavior Journal standing out as the publisher of two articles. The other journals - *Journal of Biosocial Science, Behavioral Medicine, STI Online First, J Acquir Immune Defic Syndr, BMC Infectious Diseases, Annals of Epidemiology, Health & Place*, published one article. As for the methodological aspects, ten studies used the quantitative approach, and one employed the qualitative approach. Among the quantitative studies, six used cross-sectional design, and three used cohort design.

Among the locations where the investigations were conducted, the United States of America (USA) stood out with four publications, followed by the sub-Saharan African region (two) and one publication related to the countries of Malaysia, South Africa, Ethiopia, and Colombia. That said, we observe that both developed, developing and underdeveloped countries are appropriating the knowledge of multifactorial factors that interfere directly and indirectly, as protective or risk factors in HIV transmission, which tends to favor the development of public policies more tangible with the reality and local needs.

### Structural and behavioral risk factors

In this category, the main risk factors associated with HIV infection were described and categorized according to the structural and behavioral elements of the individual^(20)^. Regarding the structural elements, economic disadvantage^(22,10)^, social disorder^(23,26)^, neighborhood characteristics^(5,22-23)^, housing instability^(24,27)^ and incarceration^(25,27)^, represented greater distribution in the syntheses of the included studies. Regarding behavioral factors, the studies pointed to transactional sex^(10,22,27)^, multiple partners^(5,25)^, substance abuse^(23,25)^, and age at first intercourse^(23,25)^ as risk factors. We used the ten studies: S1, S2, S3, S4, S5, S6, S7, S8, S9, and S10, as represented in Chart 2.

### Structural and behavioral protective factors

In this category, the main protective factors of exposure to HIV were described according to the structural and behavioral elements of the individual^(20)^. According to the synthesis of the selected studies, accessibility to voluntary testing^(4,21)^, reduced socioeconomic disadvantage, and provision of housing stability^(22,27)^ were the most frequent findings associated with structural elements. As for the behavioral elements, condom use^(2,26)^ was described in the studies as the main HIV protection factor. Eight studies were used: S1, S2, S3, S4, S5, S7, S8, and S9, as represented in Chart 3.

## DISCUSSION

The selected studies point to the growth in the last ten years of research, emphasizing risk factors in the young population. Data corroborate that young people still present risks inherent to HIV exposure regarding social and economic vulnerability factors and risk behaviors. There is a scarcity in national studies, highlighting fragility in the knowledge of practices and attitudes, as well as structural context associated with HIV infection in Brazil in the light of multilevel analysis^(1,19)^.

We emphasize that socio-structural circumstances are often associated with risk behaviors and epidemic spread^(28)^. The studies grouped in the category *structural and behavioral risk factors*, signal disorders and socioeconomic disadvantages as structural factors present in the risk association. An observational study conducted in Colombia showed significant associations between the prevalence of HIV (n=2283) by self-report related to sanitary infrastructure, housing quality, education, and access to health services, it also highlighted the higher presence of infection in young people, especially men in productive age^(29)^.

In Brazil’s southern region, another study identified increased HIV incidence in poor populations, with a high frequency of single-parent families headed by women, with socioeconomic and structural disadvantages that point to poverty and gender as factors associated with HIV feminization^(30)^.

Another relevant aspect of this study’s outcome was related to neighborhood characteristics. Research conducted in 20 countries in sub-Saharan Africa shows that the fact that young people are less geographically mobile compared to adults, with more evident permanence in their residential neighborhoods, and generally in the same social network of conviviality, makes them exceptionally vulnerable to social relations and behavioral influences oral risk transmitted by the community^(4)^.

Regarding influences, we observe the relationship to media exposure as a risk factor in the categories, a study investigating practices and risk behaviors in southern Brazil pointed to the influence of social media and its impact on sexuality with a consequent higher rate of use for unprotected sexual encounters^(31)^. Distinctly, another study pointed out in ten Brazilian cities that regarding the risk perception and the ease of HIV transmission, the information provided by the media, family members, and non-governmental organizations (NGOs) has an impact on sexual risk protection^(32)^.

In the social disparities context, the migration process can be associated. A theoretical and conceptual analysis of migration flows has shown that access to health services, as well as a disadvantage and social exclusion, are related to predicting migration and increased risk exposure. The outcomes of these correlations are associated with vulnerability to HIV^(28)^.

Associations with incarceration corroborate a study in the central-western region of Brazil, investigating the population base of prisoners. It was observed that HIV outcome with previous incarceration history was more evident in males^(33)^. Similar to data scored in the Northeast region of the same country, where HIV-positive individuals in incarceration were predominantly single, separated, and widowed, with a mean age of 31.3 years and African-American^(34)^. The research addresses incarceration as an unfavorable condition of social vulnerability, with evidence of increased incidence in the Middle East and North Africa in prisons and associations of a similar situation in the Pacific and Asia^(35-36)^.

Regarding behavioral factors, a cohort study conducted in northern Uganda found that transactional sex was associated with a higher risk of HIV infection^(37)^.

Faced with exposure, multiple partnerships are factors that are more associated with higher sexual transmissions. International research conducted with women found that the risk of contracting HIV may be higher in a partnership that involves simultaneity^(38)^, corroborating the data from a national study conducted with young people, which showed statistical significance in relation to risk behavior comparing sex and number of partners^(39)^.

Substance abuse was highlighted in a risk-ratio analysis with African-American girls using a multivariate model, in which the group with the highest exposure was associated with a higher likelihood of multiple partnerships, high alcohol and other drug use, low frequency of condom use, and history of Sexually Transmitted Infections (STIs) when compared to groups classified as lower risk^(40)^.

The age of first sexual intercourse was highlighted as a risk factor for HIV infection in research conducted in southeastern Brazil, investigating the perception of young people regarding sexual practices^(41)^, as well as in another Brazilian research, which associated HIV with the average age of 13.6 years for boys and 14.6 years for girls^(42)^. Regarding risk behavior and predictors, another investigation showed that people who started sexual life early were more likely not to use condoms in the last intercourse^(43)^.

In the category *structural and behavioral protective factors*, the practices, attitudes, habits, and routines associated with lower exposure to HIV were highlighted^(12)^. We observed that accessibility to voluntary testing as a protective factor to HIV and universal access to serological screening, including HIV are components associated with prevention and reduction in the chain of transmission. Data from a European study offering testing in emergency services associated the lower propensity of test acceptability among young people who perceived themselves to be at reduced risk of HIV or if they had been previously tested^(44)^.

Reduced socioeconomic disadvantage and stable housing correlate with study, where economic constraints are related to a limitation in seeking HIV clinical care, so it is a predictor of protection to support access to livelihood development for economic empowerment^(45)^. The unfavorable socioeconomic conditions may be influenced by a knowledge gap about HIV and AIDS, affecting mainly people with lower education level and social class^(32)^. Pre-exposure prophylaxis (PrEP) adherence studies reaffirm that being transgender, young, and having lower socioeconomic status increases the chances of low adherence to prophylaxis^(46)^.

We must highlight that stable housing can be an effective strategy at the international level to reduce the risk behaviors associated with HIV, guaranteeing housing and fixed housing permeates the precepts of providing adequacy with an impact not only in prevention but in reducing morbidity by HIV and AIDS and several other chronic diseases^(47)^.

Brazilian population-based study emphasizes socioeconomic and cultural inequalities and institutional racism associated with high vulnerability to HIV in black women since safer behavior is associated with the distribution of economic and cultural resources and social pressures, which remain in inadequate and inconsistent redistribution according to unequal gender assessment among other factors^(48)^.

Still, community support as a care network involves linkage with health services and community institutions to improve access to care strategies, in this context, evidence associates that structural interventions require community participation and mobilization^(49)^.

Adherence to condom use as a behavioral intervention still faces daily challenges in obtaining more significant impacts in the population segments^(1,49)^. During a study conducted in Brazil’s Northeast region, it was observed that non-adherence to condoms involves emotional, affective, and social dimensions, such as trust in the partner and negotiating the use, with evidence of dispensing use in sexual intercourse with fixed partners^(50)^.

Attitudes toward negotiating condom use are also associated with autonomy in sexual relations, representing a factor associated with a lower risk of exposure to HIV. Corroborating with studies, where the number of partners is often associated with risky sexual behavior, however, research shows that maintaining safe sex is more important than the association with the number of sexual partners^(39,50)^.

Sexual patterns are associated with lower exposure, a survey conducted with young people found that sexual behaviors have a tendency to a pattern when associated with condom use at first and last sexual intercourse, 71.4% of young respondents who reported condom use at first intercourse, ratified use at last intercourse^(39)^.

Other factors pointed out in the study should be emphasized by health services to develop specific approaches for a better individual perception of risk exposure. The multilevel model presented the behavioral and structural aspects as important for understanding the diversity of needs and demands that should be translated into behavioral and structural interventions in HIV risk reduction.

### Study Limitations

We highlight the scarcity of studies in the national literature that involve the multilevel model in identifying structural and behavioral elements for sample eligibility, which limited the discussion of the data in comparing the findings in Brazilian youth and adults.

### Contributions to the Area

Knowledge of risk factors by nursing and health professionals can lead to better applicability of strategies with an emphasis on behavioral and structural measures. Creating proposals through HIV protection and risk factors may stimulate specific approaches, providing support and promoting the perception of HIV risk exposure.

This study may also contribute to further research in Brazil, focusing on a multilevel model for better sample representativeness and, consequently, results applicable according to the behavioral and/or structural risk context inherent to the individual.

## CONCLUSIONS

The applicability of the multilevel model in risk factor research studies allowed the identification of structural and behavioral elements associated with HIV infection. Economic disadvantage, neighborhood characteristics, incarceration, and housing instability were classified as structural risk factors. Transactional sex, multiple partners, substance abuse, and age at first intercourse as behavioral risk factors for HIV, reduced socioeconomic disadvantage, provision of housing stability, and condom use were associated with protective factors to HIV exposure.

These aspects must be considered for the production of care, which attends to the singularities according to structural and behavioral elements involved in the increased vulnerability of young people and adults to HIV infection.

This research points to the importance of developing differentiated interventions by the health team, to increase risk protection against HIV. The data found in this study shows that risk attitudes and practices are not limited and should not be seen in isolation. In this perspective, the performance of nursing and health professionals in implementing interventions that involve the social structure of the individual as well as behavioral practices should have research potential to be explored.

## References

[1] Joint United Nations Programme on HIV/Aids (UNAIDS) (2021). Estimates and additional data are [Internet].

[2] Ministério da Saúde (BR) (2020). Boletim Epidemiológico HIV/Aids 2020 [Internet].

[3] Ward-Peterson M, Fennie K, Mauck D, Shakir M, Cosner C, Bhoite P (2018). Using multilevel models to evaluate the influence of contextual factors on HIV/AIDS, sexually transmitted infections, and risky sexual behavior in sub-Saharan Africa: a systematic review. Ann Epidemiol.

[4] Uchudi J, Magadi M, Mostazir M. (2012). A multilevel analysis of the determinants of high-risk sexual behaviour in sub-Saharan Africa. J Biosoc Sci.

[5] Tomita A, Vandormael AM, Bärnighausen T, Oliveira T, Tanser F. (2017). Social disequilibrium and the risk of HIV acquisition: a multilevel study in Rural KwaZulu-Natal Province, South Africa. J Acquir Immune Defic Syndr.

[6] Jonas K, Crutzen R, Borne VDB, Sewpaul R, Reddy P. (2016). Teenage pregnancy rates and associations with other health risk behaviours: a three-wave cross-sectional study among South African school-going adolescents. Reprod Health.

[7] Salazar LF, Bradley ELP, Younge SN, Daluga NA, Crosby RA, Lang DL (2010). Applying ecological perspectives to adolescent sexual health in the United States: rhetoric or reality?. Health Educ Res.

[8] Costa ACPJ, Lins AG, Araújo MFM, Araújo TM, Gubert FA, Vieira NFC. (2013). Vulnerability of adolescent students to STD / HIV in Imperatriz - Maranhão. Rev Gaúcha Enferm.

[9] Olding M, Enns B, Panagiotoglou D, Shoveller J, Harrigan R, Barrios R (2017). A historical review of HIV prevention and care initiatives in British Columbia, Canada: 1996-2015. J Int AIDS Soc.

[10] Magadi M, Desta M. (2011). A multilevel analysis of the determinants and cross-national variations of HIV seropositivity in sub-Saharan Africa: evidence from the DHS. Health Place.

[11] Puente-Palacio KE, Laros JA. (2009). Multilevel analysis: contributions to studies investigating the effects of social contexto on individual behavior. Estud Psicol.

[12] Hargreaves JR, Delany-Moretlwe S, Hallett TB, Johnson S, Kapiga S, Bhattacharjee P (2016). The HIV prevention cascade: integrating theories of epidemiological, behavioural, and social science into programme design and monitoring. Lancet HIV.

[13] Santos JLG, Ali Pestana AL, Guerrero P, Meirelles BSH, Erdmann AL. (2013). Práticas de enfermeiros na gerência do cuidado em enfermagem e saúde: revisão integrativa. Rev Bras Enferm.

[14] Lourenço TM, Lenardt MH, Kletemberg DF, Seima MD, Tallmann AEC, Neu DKM. (2012). Capacidade funcional no idoso longevo: uma revisão integrativa. Rev Gaúcha Enferm.

[15] Sousa LMM, Firmino CF, Marques-Vieira CMA, Severino SSP, Pestana CFC. (2018). Scientific literature reviews: types, methods and applications in nursing. Rev Port Enferm Reabil.

[16] Donato H, Donato M. (2019). Stages for Undertaking a Systematic. Acta Med Port.

[17] Salameh J, Bossuyt PM, McGrath TA, Thombs BD, Hyde CJ, Macaskill P (2020). Preferred reporting items for systematic review and meta-analysis of diagnostic test accuracy studies (PRISMA-DTA): explanation, elaboration, and checklist. BMJ.

[18] Ouzzani M, Hammady H, Fedorowicz Z. (2016). Rayyan: a web and mobile app for systematic reviews. Syst Rev.

[19] World Health Organization (WHO) (2013). HIV and adolescents: guidance for HIV testing and counseling and care for adolescents living with HIV: recommendations for a public health approach and considerations for policy-makers and managers [Internet].

[20] Price JT, Rosenberg NE, Vansia D, Phanga T, Bhushan NL, Maseko B (2018). Predictors of HIV, HIV Risk Perception, and HIV Worry among Adolescent Girls and Young Women in Lilongwe, Malawi. Acquir Immune Defic Syndr.

[21] Burch WJ, Hart GJ, Lim SH (2018). A qualitative study of young men who have sex with men and multilevel factors related to HIV Risks in Malaysia. AIDS Educ Prev.

[22] Bauermeister J, Eaton L, Stephenson R. (2016). A multilevel analysis of neighborhood socioeconomic disadvantage and transactional sex with casual partners among young men who have sex with men living in Metro Detroit. Behav Med.

[23] Haley DF, Kramer MR, Adimora AA, Haardörfer R, Wingood GM, Ludema C (2017). Relationships between neighborhood characteristics and current STI status among HIV-infected and HIV-uninfected women living in the Southern USA: a cross-sectional multilevel analysis. Sex Transm Infect.

[24] Hailu BA, Tadese F, Bogale GG, Molla A, Miherety BA, Beyene J. (2020). Spatial patterns and associated factors of HIV Soropositivity among adults in Ethiopia from EDHS 2016: a spatial and multilevel analysis. BMC Infect Dis.

[25] Adimora AA, Schoenbach VJ, Taylor EM, Khan MR, Schwartz RJ, Miller WC. (2013). Sex ratio, poverty, and concurrent partnerships among men and women in the United States: a multilevel analysis. Ann Epidemiol.

[26] Miller RL, Strzyzykowski T, Lee KS, Chiaramonte D, Acevedo Polakovich I, Spring H. (2018). Structural efects on HIV risk among youth: a multi level analysis. AIDS Behav.

[27] Stoner MCD, Haley DF, Golin CE, Adimora AA, Pettifor A. (2019). The relationship between economic deprivation, housing instability and transactional sex among women in North Carolina (HPTN 064). AIDS Behav.

[28] Sacramento O. (2019). Migration policy, structural violence and HIV/AIDS. Espaço Aberto.

[29] Tovar-Cuevas LM, Arrivillaga-Quintero M. VIH (2011). / SIDA y determinantes sociales estructurales en municipios del Valle del Cauca-Colombia. Rev Gerenc Polit Salud [Internet].

[30] Ceccon RF, Meneghel SN, Hirakata NV. (2014). Women with HIV: gender violence and suicidal ideation. Rev Saúde Pública.

[31] Nogueira CF, Cerdeira CD, Prado AC, Dias RPCS, Silva RBV, Vertêlo PC (2020). Profile of people living with HIV at a reference center in contagious and infectious diseases in Belo Horizonte (MG, Brazil). Rev Med Saúde[Internet].

[32] Gomes RRFM, Ceccato MGB, Kerr LRFS, Guimarães MDC. (2017). Fatores associados ao baixo conhecimento sobre HIV/AIDS entre homens que fazem sexo com homens no Brasil. Cad Saúde Pública.

[33] Sgarbi RVE, Carbone AdSS, Paião DSG, Lemos EF, Simionatto S, Puga MAM (2015). A Cross-Sectional Survey of HIV Testing and Prevalence in Twelve Brazilian Correctional Facilities. PLoS One.

[34] Sousa KAA, Araújo TME, Teles SA, Rangel EML, Nery IS. (2017). Factors associated with HIV prevalence in a prison population. Rev Esc Enferm USP.

[35] Golrokhi R, Farhoudi B, Taj L, Pahlaviani FG, Mazaheri-Tehrani E, Cossarizza A (2018). HIV Prevalence and Correlations in Prisons in Different Regions of the World: a review article. Open AIDS J.

[36] El Maerrawi I, Carvalho HB (2015). Prevalence and risk factors associated with HIV infection, hepatitis and syphilis in a state prison of São Paulo. Int J STD AIDS.

[37] Karamagi E, Sensalire S, Nabwire J, Byabagambi J, Awio AO, Aluma G (2018). Quality improvement as a framework for behavior change interventions in HIV-predisposed communities: a case of adolescent girls and young women in northern Uganda. AIDS Res Ther.

[38] Maughan-Brown B, Kenyon C, Lurie MN. (2014). Partner age differences and concurrency in South Africa: implications for HIV-infection risk among young women. AIDS Behav.

[39] Rizzon BB, Souza VB, Madeira K, Machado LV, Magalhães M. (2021). Comportamento de risco para infecções sexualmente transmissíveis em estudantes do ensino médio. Femina [Internet].

[40] Danielson CK, Walsh K, McCauley J, Ruggiero KJ, Brown JL, Sales JM (2014). HIV-related sexual risk behavior among African American adolescent girls. J Womens Health (Larchmt).

[41] Spindola T, Santana RSC, Costa CMA, Martins ERC, Moerbeck NT, Abreu TO. (2020). It won’t happen: college students’ perception of sexual practices and vulnerability to sexually transmitted infections. Rev enferm UERJ.

[42] Chaves CS, Rouberte ESC, Costa EC, Moura ADA, Rodrigues VC, Souza ALS (2021). Vulnerabilidade às infecções sexualmente transmissíveis de adolescentes privados de liberdade. BJHR.

[43] Moreira LR, Dumith SC, Paludo SS. (2018). Condom use in last sexual intercourse among undergraduate students: how many are using them and who are they?. Ciênc Saúde Colet.

[44] d’Almeida WK, Pateron D, Kierzek G, Renaud B, Semaille C, Truchis P (2013). Understanding Providers’ Offering and Patients’ Acceptance of HIV Screening in Emergency Departments: a multilevel analysis. PLoS ONE.

[45] World Health Organization (WHO) (2016). Consolidated guidelines on HIV prevention, diagnosis, treatment and care for key populations - 2016 update [Internet].

[46] Zucchi EM, Grangeiro A, Ferraz D, Pinheiro TF, Alencar T, Ferguson L (2018). Da evidência à ação: desafios do Sistema Único de Saúde para ofertar a profilaxia pré-exposição sexual (PrEP) ao HIV às pessoas em maior vulnerabilidade. Cad Saúde Pública.

[47] Adimora AA, Auerbach JD. (2010). Structural interventions for HIV prevention in the United States. J Acquir Immune Defic Syndr.

[48] Santos NJS. (2016). Mulher e negra: dupla vulnerabilidade às DST/HIV/aids. Saude Soc.

[49] Krishnaratne S, Hensen B, Cordes J, Enstone J, Hargreaves JR. (2016). Interventions to strengthen the HIV prevention cascade: a systematic review of reviews. Lancet HIV.

[50] Garcia EC, Costa IV, Oliveira RC, Silva CRL, Góis ARS, Abrão FMS. (2021). Social representations of adolescents about HIV/AIDS transmission in sexual relations: vulnerabilities and risks. Esc Anna Nery.

